# Neural Signals Evoked by Stimuli of Increasing Social Scene Complexity Are Detectable at the Single-Trial Level and Right Lateralized

**DOI:** 10.1371/journal.pone.0121970

**Published:** 2015-03-25

**Authors:** Carlos P. Amaral, Marco A. Simões, Miguel S. Castelo-Branco

**Affiliations:** 1 IBILI—Institute for Biomedical Imaging in Life Sciences, Faculty of Medicine, University of Coimbra, Coimbra, Portugal; 2 ICNAS, Brain Imaging Network of Portugal, Coimbra, Portugal; Centre de Neuroscience Cognitive, FRANCE

## Abstract

Classification of neural signals at the single-trial level and the study of their relevance in affective and cognitive neuroscience are still in their infancy. Here we investigated the neurophysiological correlates of conditions of increasing social scene complexity using 3D human models as targets of attention, which may also be important in autism research. Challenging single-trial statistical classification of EEG neural signals was attempted for detection of oddball stimuli with increasing social scene complexity. Stimuli had an oddball structure and were as follows: 1) flashed schematic eyes, 2) simple 3D faces flashed between averted and non-averted gaze (only eye position changing), 3) simple 3D faces flashed between averted and non-averted gaze (head and eye position changing), 4) animated avatar alternated its gaze direction to the left and to the right (head and eye position), 5) environment with 4 animated avatars all of which change gaze and one of which is the target of attention. We found a late (> 300 ms) neurophysiological oddball correlate for all conditions irrespective of their complexity as assessed by repeated measures ANOVA. We attempted single-trial detection of this signal with automatic classifiers and obtained a significant balanced accuracy classification of around 79%, which is noteworthy given the amount of scene complexity. Lateralization analysis showed a specific right lateralization only for more complex realistic social scenes. In sum, complex ecological animations with social content elicit neurophysiological events which can be characterized even at the single-trial level. These signals are right lateralized. These finding paves the way for neuroscientific studies in affective neuroscience based on complex social scenes, and given the detectability at the single trial level this suggests the feasibility of brain computer interfaces that can be applied to social cognition disorders such as autism.

## INTRODUCTION

Investigating the sensitivity of neurophysiological responses to complex social scenes is now becoming an increasingly recognized topic in affective neuroscience [1,2]. It is also of paramount importance in important fields such as developmental neuroscience [3] and autism research, where complex social attention deficits are present [2,4]. Moreover, if these responses could be studied at the single or near single-trial level, this might pave the way to develop brain computer interfaces to train social cognition deficits in these disorders, which are characterized by deficits in social attention. The problems associated with low signal to noise of neurophysiological responses are now overcome by advanced statistical classification methods that can classify these signals at the single-trial level [5,6].

Attention to social stimuli such as faces has often been studied with oddball paradigms which use simple face presentation as target stimuli and the average of many responses for the analysis (e.g., [7–10]). As an example, the P300 oddball signal is a well-known neural signature of attention processes for detection of rare items in a series of distinct stimuli types. The P300 has been classically reported as an enhanced positive-going component with a latency of about 300 milliseconds (ms) and normally a scalp distribution over the midline electrodes (for a review see [11–13]).

The main goal of this manuscript was to study attention to social complex stimuli and scenes at the single-trial level, and the relevance of hemispheric laterality in this process. This is an important qualitative step, because we focus on single or few trials in addition to average neurophysiological responses. The suitability of oddball paradigms for such single-trial analyses, has been empirically proven and is well documented in the literature. The reason for its use is that it is possible to quickly “calibrate” and model the P300 in individual subjects and use it in statistical classification approaches [5,6]. Conflict paradigms or attentional-bias paradigms have not yet been proven to be suitable for single-trial analyses, unlike P300 approaches. Accordingly, P300 based oddball paradigms are often used in brain-computer interfaces (BCI) which are systems that allow individuals to communicate without having to use verbal or motor means of communication [14–17]. The fact that P300 is a robust signal that can be identified even at single-trial level makes it a favourable neurophysiological component to provide good communication speeds for BCIs. Wang and colleagues in [18] were able to achieve an information transfer rate of 12 characters per minute with this type of paradigm, which is very significant in this field. These approaches do therefore take advantage of the recent progress in statistical classification methods (for review see [19]) to identify P300 waveforms even at the single-trial level (e. g., [6,20]).

The usage of faces as target of attention was already successful in oddball-based BCIs [21–23] and has also been used to study healthy social cognition [24] and disorders such autism [25,26], prosopagnosia [27] and social phobia [28]. Moreover, a few studies aiming at BCI applications tried to integrate three-dimensional (3D) stimuli in oddball paradigms [29–31]. They showed that it is possible to measure a P300 response to 3D stimuli though none used realistic or complex social scenes as targets of attention.

The eyes are a powerful route of non-verbal information and draw most of our attentional resources during social interactions. They can be used to determine the focus of someone's attention, which is called joint attention. This ability is already established very early in development (for review see [32]) which indicates the high relevance this kind of gaze processing occupies in the human evolutionary process. Gaze shifts of others towards any point in the environment can trigger a reflexive redirection of one's own attentional focus [33,34]. Thus, we believe that this reflexive attentional process can be studied in terms of the mechanisms involved in novelty processing embedded in oddball paradigms. Therefore, we envisaged the introduction of complex social scenes containing non-natural (flashed) or natural eye/head-gaze shifts as target of attention in oddball paradigms.

Several studies have already described the involvement of the superior temporal sulcus (STS) in the processing of relevant and familiar types of biological motion such as human body motion [35,36], expression of emotions [37–39], facial motion due to speech production [40,41], or in complex scenes such as movies [42,43]. Additionally, this region have been shown to respond to natural images of facial motion [44]. Taken together, such studies suggest that initial analysis of social cues occurs in the STS region, which is in a privileged anatomical location to integrate information derived from both the ventral “what” and the dorsal “where” visual pathways [45]. Hein and Knight in [46] agreed that the function of STS depends largely upon the co-activations of connected areas. On the other hand, Haxby et al. in [47] postulate that the posterior STS is responsible for the processing of quickly changing social features, such as facial expressions. Nummenmaa & Calder in [48] believe that the posterior STS is related to the processing of the intentionality of others’ actions. Lahnakoski et al. in [49] suggested that the posterior STS region is functionally tightly coupled with other brain regions and might work as a convergence (integration) point of social information processed in other functionally connected sub-systems.

To our knowledge, studies of complex social cognition close to the single-trial level have not been attempted from the cognitive neuroscience point of view. We based the current study on the introduction of hierarchically complex and realistic stimuli with social content. In this way we could dissect the cognitive networks underlying normal attention to stimuli of complex social significance. We believe, as Kingstone proposed in [50], that the study of the neural correlates of increasingly complex representations of social interactions can provide critical insights into the nature of cognitive processing in the domain of social attention. Furthermore, Mattout in [51]highlighted the strong need for experiments that could help identify realistic and efficient models of social interactions that BCI could then use to instantiate more productive interactions between an adaptive machine and a patient. If one could detect the neural signals related to complex social cognition processing at the single-trial level, it would potentially pave the way for the use of these kind of stimuli in future approaches of cognitive training in diseases of social cognition such autism. An interesting and updated review about the use of innovative computer technology for the development of social skills to individuals [52] reveals the promising potential of this type of approach, in particular if realistic scenes are used. The studies mentioned in this review reported significant improvements in the addressed social skills, however, altogether, the same studies did not verify the transfer of these skills to more realistic and meaningful contexts. On the other hand, some other studies have in fact explored methods for systematically teaching joint attention to children with autism [53–55]. These studies included embedding motivating social interactions into the interventions, which effectively improved children’s social competences. However, the infrequent implementation of the protocols compromised the carryover after the end of the interventions. Thereby, the use of BCI interfaces that provide detection of complex social cognition related neural signals would enable the use of structured, well-controlled, realistic and immersive social interactions in computerized systems. This would in turn facilitate the repetition of the interventions as many times as required.

Thus, we directly attempted to test the lateralization and detect the neurophysiological correlates of attention to complex social stimuli at a single-trial level as a way to prove the usability of this concept in BCI applications.

## METHODS

### Ethics Statement

This study and all the procedures were reviewed and approved by the Ethics Commission of the Faculty of Medicine of the University of Coimbra (Comissão de Ética da Faculdade de Medicina da Universidade de Coimbra) and was conducted in accordance with the declaration of Helsinki. All participants were recruited from our database of voluntary participants, with no monetary compensation. All of them agreed and signed a written informed consent.

### Participants

All participants (n = 17, 11 males, 6 females, average age 22.8 years (SD = 4.1), range 20–33 years) had normal or corrected-to normal vision and no history of neurological disorders nor any other major health problems. All participants were naive regarding the purpose of the study. Participants took part in EEG recordings during five different experimental paradigms.

### Experimental paradigms

We constructed five oddball experimental paradigms using the Vizard Virtual Reality Toolkit, from WorldViz. They ranged from simple flashing stimulus paradigms to realistic animations of human models as targets for focus of attention. The flashing paradigms were labelled as follows: ‘Flashed Schematic Eyes’, ‘Flashed Face—Eye position change’, ‘Flashed Face—Eye and Head position change’ (flashing paradigms). The paradigms with animations as target of attention were labelled as: ‘Animated 3D body—gaze change in 1 avatar’ and ‘Animated 4 avatar environment—gaze change in 4 avatars’.

**Fig 1:**
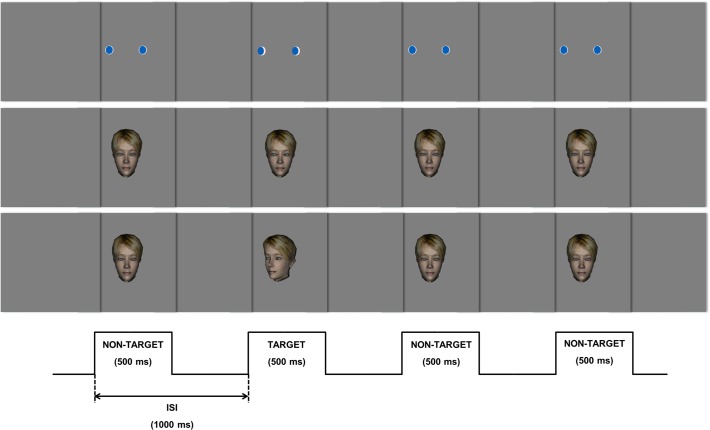
Schematic illustration of the 3 flashing oddball paradigms. Top—‘Flashed Schematic Eyes’ paradigm: The subjects were asked to count the occurrence of slightly rotated balls. Middle—‘Flashed Face—Eye position change’ paradigm: the target event is a change in the direction of the eyes. Bottom—‘Flashed Face—Eye and Head position change’: The target event is the slight head rotation.

Flashing paradigms description (Fig. 1):

- **‘Flashed Schematic Eyes’**: The non-target events of this paradigm consisted in the appearance of two 3D models of “balls” (resembling eyes) in a grey background screen, during 500 ms. In the target event the balls appeared slightly rotated in relation to the position it had in the non-target events. The task was to mentally count the occurrence of these target events;
- **‘Flashed Face—Eye position change’**: The non-target events of this paradigm consisted in the appearance of a 3D model of a face in a grey background screen, “looking” to the participant during 500 ms. In the target event the face appeared with the eyes gazing to its right (left of the participant). The task was to mentally count how many times the face appeared with the eyes gazing to participants’ left;
- **‘Flashed Face—Eye and Head position change’**: The non-target events of this paradigm consisted in the appearance of a 3D model of a face looking to participant during 500 ms. In the target event the face appeared facing towards the left side. The participant task was to mentally count the times the face appeared facing to participants’ left.

**Fig 2:**
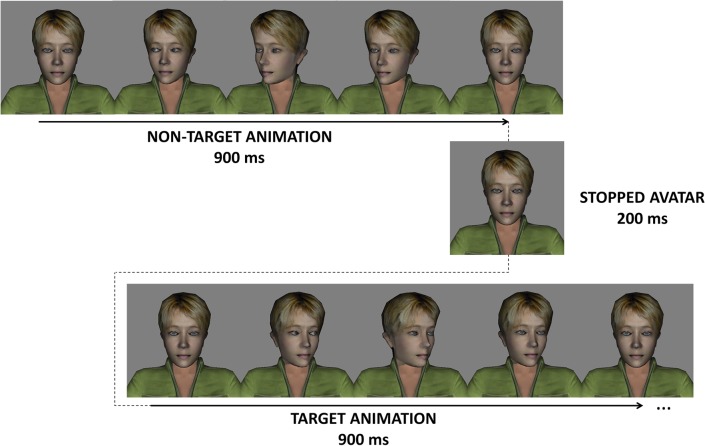
‘Animated 3D body—gaze change in 1 avatar’ paradigm. The participants were instructed to pay attention to the turning of the head of the avatar to the left of the participant. Non-target animation is the rotation of the head to the right of the participant.

**Fig 3:**
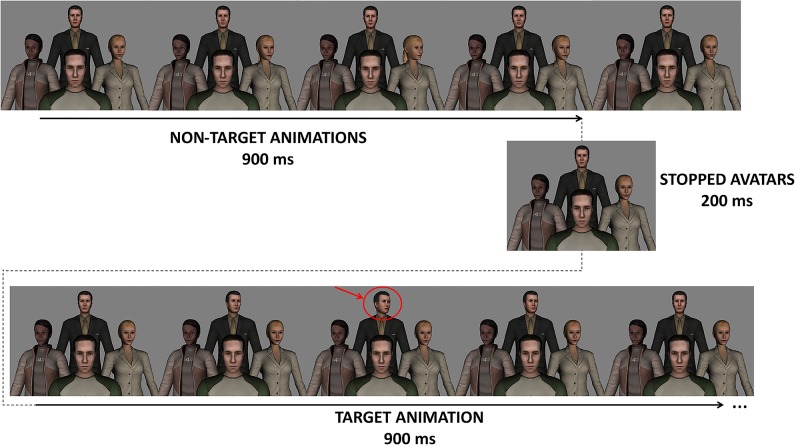
‘Animated 4 avatar environment—gaze change in 4 avatars’ paradigm. The target of attention is the animation of top avatar. The task was to count how many times the top avatar averted its gaze.

Animated paradigms description:

- **‘Animated 3D body—gaze change in 1 avatar’**: One 3D model of a human-like avatar is facing the participant (presented from the shoulders up), in a scenario with a grey background. The events are the animation of this avatar. The non-target event is the rotation of avatar’s head to the left side (continuous realistic animation that lasted 900 ms). The target event was the rotation of the head to the right side (also a continuous realistic animation of 900 ms). The task of the participant was to mentally count “*how many times the person looks to its right”* (Fig. 2).
- **‘Animated 4 avatar environment—gaze change in 4 avatars’**: Four different avatars are arranged in diamond, in a scenario with a grey background. The events consisted in the rotation of the head of one of the four avatars to the right (continuous realistic animation that lasted 900 ms). The target event was the rotation of the head of the diamond’s top edge avatar, to the right (see Fig. 3). The task here was to mentally count “*how many times the person in the top looks to its right side”*.

We ran 10 blocks of 50 events for each paradigm. We allowed the participant to rest at each 5 blocks. In all the paradigms the target events were displayed randomly among the non-target. In each block the number of target events was 10, which gives an occurrence probability of 1/5. Two target events never occurred consecutively. In the ‘Animated 4 avatar environment—gaze change in 4 avatars’ paradigm, the same avatar never turned its head two times consecutively. The stimulus onset asynchrony (SOA) of the flashing paradigms was 1000 ms and the inter-stimulus interval (ISI) was 500 ms. In the animated paradigms the SOA was 1100 ms and the time between the animations of the avatars was 200 ms (thus animation time is 900 ms). The animation time of these two paradigms is the balance between the time needed to maintain a realistic movement of the avatar head and the need to maintain the time as small as possible to avoid uncomfortable total experiment times.

We planned to introduce hierarchical social scene complexity in these paradigms to uncover the degree of complexity that can be introduced in oddball paradigms such that the neural correlates for attention to social stimuli can still be detected at single-trial level. The social scene complexity of these paradigms was organized according to a matrix which ordered three objective parameters present in the scene. This matrix takes into account the number of items in the scene (including multipart social objects), elements that define the trajectory of social object and presence of movement. See Table 1.

**Table 1:** Matrix describing ordinal criteria for paradigms social scene complexity. These criteria define the hierarchy of social complexity of the paradigms.

Stimuli	Amount of items in the scene (including multipart social objects)	Elements that define the trajectory of social object	Movement	Overall social scene complexity
Flashed Schematic Eyes	* (2 balls)	* (2 balls)	*	***
Flashed Face—Eye position change	** (face)	* (2 eyes)	*	****
Flashed Face—Eye and Head position change	** (face)	** (eyes and face)	*	*****
Animated 3D body—gaze change in 1 avatar	*** (face and upper chest)	** (eyes and face)	**	*******
Animated 4 avatar environment—gaze change in 4 avatars	**** (4 faces and upper bodies)	** (eyes and face)	**	********

After each block the participants were asked how many target events they counted.

### Data Acquisition

Participants were sat at about 60 centimetres from the screen (HP L1710 17-inch LCD Monitor; frame rate of 60 Hz), and the EEG was recorded using a Brain Products Package.

The individuals scalp was first cleaned using abrasive gel and then the actiCAP cap was placed on their heads. The data was recorded from 16 Ag/AgCl active electrodes (Brain Products), placed in Fp1, F3, Fz, F4, FCz, C3, Cz, C4, T4, P7, P3, Pz, P4, P8, O1, and O2 locations according to the international 10–20 standard system. The ground electrode was placed at AFz position and the reference electrode at T3 position. Their impedance was kept under 10 kΩ. The electrodes were connected directly to the 16 channels Brain Products V-Amp Amplifier and sampled at 1000 Hz. EEG data was recorded using the Brain Products Brain Recorder software with notch filter at 50 Hz, while the stimuli were presented to the subjects. For each paradigm the individuals were informed about the respective tasks. Each experimental procedure (preparation + 5 paradigms) took around 70 minutes to complete.

### Data analysis

We performed an off-line analysis with Brain Vision Analyzer 2 from Brain Products software. The average of T4 and T3 channels was used to form a new reference as a way to simulate as close as possible the linked ears reference due to the proximity of these electrodes to the ears. This averaged re-reference was applied to all the remaining electrodes.

The data were filtered with a low pass filter at 30 Hz (24 dB/octave) and a high pass filter at 0,16 Hz (24 dB/octave). The data segmentation was based in the SOA of each paradigm. For the flashed stimuli paradigms the segmentation was performed in epochs of 1100 ms with a 100 ms pre-stimulus interval and a 1000 ms post-stimulus interval. For the animated stimuli paradigms the segmentation was performed in epochs of 1200 ms with a 100 ms pre-stimulus interval and an 1100 ms post-stimulus interval. Segments contaminated with eye blinks or excessive muscular activity were excluded from further analysis. Artefact rejection was set at 100 microvolts (μV). Both Target and Non-target conditions yielded more than 60 segments after artefact rejection for each condition. Next, a DC trend correction was performed in each individual segment using the first 100 ms at segment start and the last 100 ms at segment end [56]. A baseline correction procedure was done using the first pre-stimulus 100 ms.

An average of the target and non-target segments was then calculated and a conventional P300 analysis was performed. For this purpose the largest positive peak occurring within 250–800 ms that increases in amplitude from Frontal to Parietal scalp areas was identified as the P300 peak. We selected the Non-Target waveforms amplitudes with the same latency as the P300 peak to make the amplitude comparisons.

General statistical analysis was performed with the software IBM SPSS Statistics 19 (SPPS, Inc.) after verifying normality assumptions, with the significance level set at 0.05 level. If the normality assumption were met we performed a 3 (area: frontal (F3, Fz, F4), central (C3, Cz, C4), parietal (P3, Pz, P4)) × 3 (location relative to midline: left (F3, C3, P3), midline (Fz, Cz, Pz) and right (F4, C4, P4)) × 2 (stimulus type: non-target, target) repeated measures ANOVA for all paradigms and a more detailed 3 (area: frontal, central, parietal) × 2 (location relative to midline: left, right) repeated measures ANOVA of the target averaged amplitudes. The *post-hoc* tests were then performed with Bonferroni correction. When the normality assumptions were not met we performed the Friedman tests and the post-hoc Wilcoxon signed-rank tests with Bonferroni correction.

The automatic classification was performed in MathWorks Matlab, using the PRTools toolbox [57]. The EEG data was split in segments from 200 ms to 800 ms after stimulus onset. Each epoch was decimated by a factor of 20 and the 16 channels were joined, forming the feature vector. Classification of the event as target or non-target was performed using a Support Vector Machine (SVM) classifier with a polynomial kernel of one degree [58]. Data were classified using several values of averaged ERPs (1, 2, 3, 4, 5, 10 and 15). Classification performance measures for target detection were obtained through leave one out 6-fold cross validation. The metrics used were accuracy—(*TP + TN*) / *N*, specificity—*TN* / (*TN + FP*), sensitivity—*TP* / (*TP + FN*) and balanced accuracy—(*SP + SS*) / *2*, having TP, TN, FP, FN, SP, SS and N as True Positives, True Negatives, False Positives, False Negatives, Specificity, Sensitivity and total number of events, respectively. The unbalanced nature of the data set (the non-target segments are four times more than the target ones, because of the different occurrence probability) makes the balanced accuracy the more reliable metric for assessing the classifier performance.

Permutation tests were performed in order to evaluate the statistical significance of the classification’s results, following the permutation-based p-value definition presented in [59]:

**Permutation-based p-value—**Let D* = {*D*’_1_, …, *D*’_*k*_} be the set of *k* randomized versions of the data matrices, $D={\left\{\left(X_{i},y_{i}\right)\right\}}_{i=1}^{n}$, where *X*_*i*_ are the observations of a series of features, and *y*_*i*_ the class labels associated to each observation, sampled from a given null distribution. The empirical p-value for the function learned by the classification algorithm, *f*, is calculated by

$$p=\frac{\left|\left\{D^{\prime }\in D^{*}:e\left(f,D^{\prime }\right)\leq e\left(f,D\right)\right\}\right|+1}{k+1},$$

*e* being the error function, which is the ratio of wrong classified observations. This *p* value represents the fraction of randomized versions where the classifier had better performance in the random labelled data than in the original data. If the *p*-value is small enough the null hypothesis is rejected. In this case the null hypothesis was that the classifier is performing at the chance level.

## RESULTS

Statistical analysis revealed a significant main effect for stimulus type in all paradigms. The lateralization analysis revealed significant main effects of location relative to midline only in the target peaks of the animated paradigms, being significantly higher at right electrode sites (for control analyses concerning gaze directions see below). Regarding the area effects, the amplitudes of P300 peaks were generally significantly higher at parietal sites in all paradigms. Latency analysis showed that latencies were significantly higher at frontal and central sites comparing to parietal sites in the less salient condition (‘Flashed Face—Eye position change’ paradigm). Detailed statistical results are shown next.

Concerning the significant main effect of stimulus type that emerged in all conditions it can be summarized as follows: ‘Flashed Schematic Eyes’—F(1,16) = 73.2, *p* < 0.0001; ‘Flashed Face—Eye position change’—F(1,16) = 25.9, *p* < 0.0001; ‘Flashed Face—Eye and Head position change’—F(1,16) = 25.5, *p* < 0.0001; ‘Animated 3D body—gaze change in 1 avatar’—F(1, 16) = 52.2, *p* < 0.0001; ‘Animated 4 avatar environment—gaze change in 4 avatars’—F(1,16) = 110.6, *p* < 0.0001. Accordingly, the waveform amplitudes of P300 to target stimuli were significantly higher than the amplitudes of the Non-Target waveforms for all the paradigms (see Table 2).

**Table 2:** Means (SEM) of the non-target and target peak amplitude (in microvolts) responses for the different paradigms.

Stimuli	Non-target	Target
Flashed Schematic Eyes	0.55 (0.23)	6,15 (0,24) ***
Flashed Face—Eye position change	1,61 (0,33)	5,31 (0,27) ***
Flashed Face—Eye and Head position change	1,18 (0,30)	5,81 (0,35) ***
Animated 3D body—gaze change in 1 avatar	1,69 (0,16)	6,72 (0,26) ***
Animated 4 avatar environment—gaze change in 4 avatars	-0,30 (0,07)	4,15 (0,20) ***

^***^ P-value < 0.0001.

**Fig 4:**
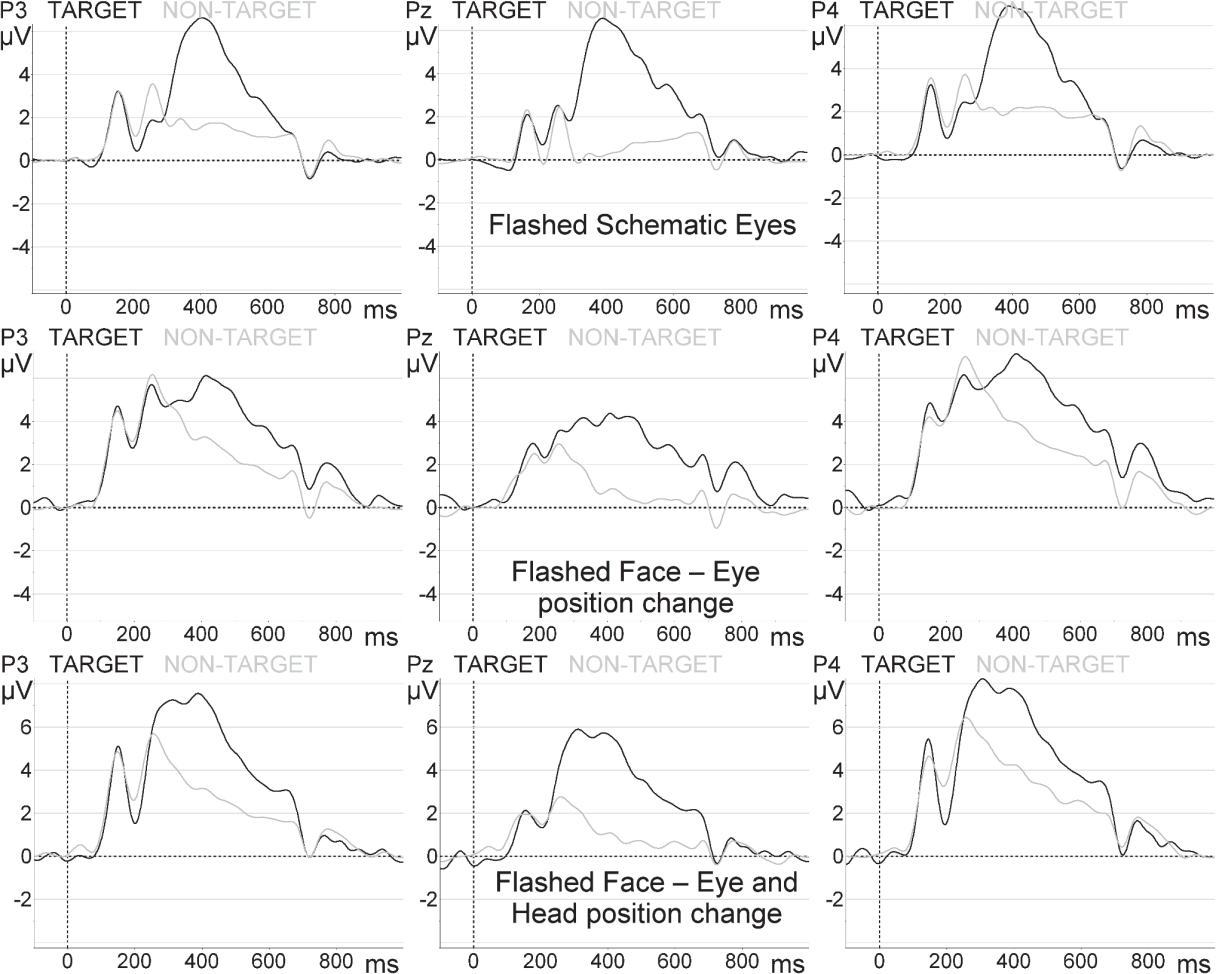
Target and non-target grand-average ERP waveforms at parietal sites. Top: ‘Flashed Schematic Eyes’ paradigm. Middle: ‘Flashed Face—Eye position change’. Bottom: ‘Flashed Face—Eye and Head position change’.

Grand-average responses in parietal sites of the flashing paradigms are shown in Fig. 4.

**Fig 5:**
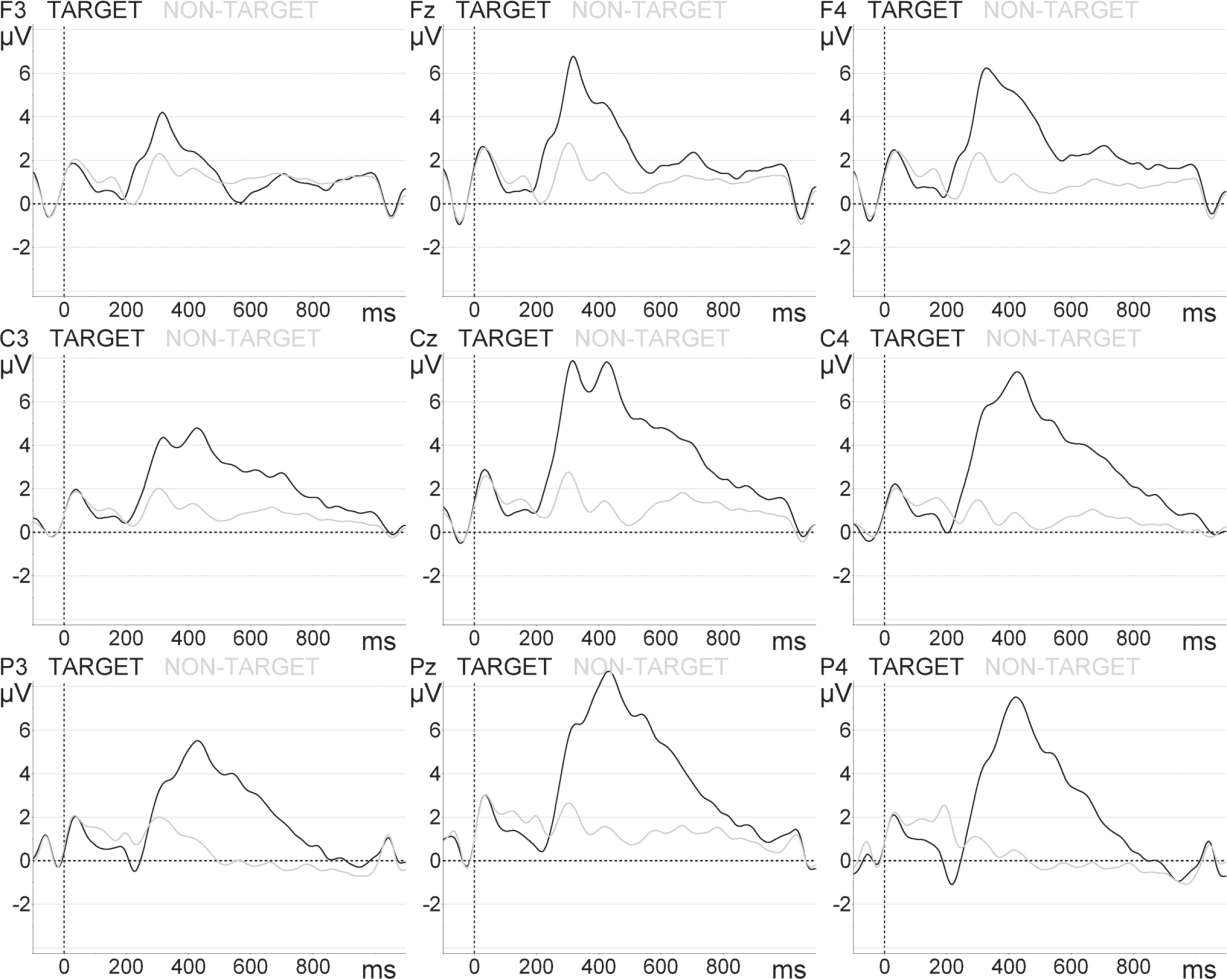
Target and non-target grand-average ERP waveforms for the ‘Animated 3D body—gaze change in 1 avatar’ paradigm.

**Fig 6:**
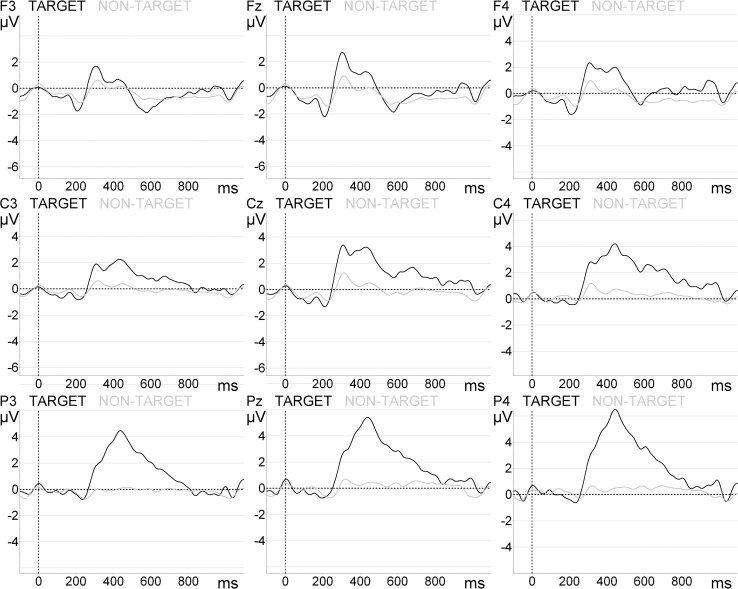
Target and non-target grand-average ERPs waveforms for the ‘Animated 4 avatar environment—gaze change in 4 avatars’ paradigm.

More detailed analysis of the target averaged amplitudes revealed marginal effects of area (F(2, 32) = 3.513, *p* = 0.073) and location (F(1, 16) = 4.439, *p* = 0.051) for the ‘Flashed Schematic Eyes’ paradigm. Concerning the ‘Flashed Face—Eye position change’ paradigm we observed area effects (F(2, 32) = 18.953, *p* < 0.0001), but not location (F(1, 16) = 1.457, *p* = 0.245). Areal effects for the ‘Flashed Face—Eye and Head position change’ paradigm were also detected (F(2, 32) = 16.857, *p* < 0.0001), contrary to location (F(1, 16) = 4.965, *p* = 0.321). The area effects were also significant for the ‘Animated 4 avatar environment—gaze change in 4 avatars’ paradigm (F(2, 32) = 19.350, *p* < 0.0001), but not for the ‘Animated 3D body—gaze change in 1 avatar’ condition. Still, as visible in Fig. 5 and Fig. 6, a location effect was indeed found in both animated paradigms: ‘Animated 3D body—gaze change in 1 avatar’—F(1, 16) = 20.518, *p* < 0.0001); ‘Animated 4 avatar environment—gaze change in 4 avatars’—F(1, 16) = 14.549, *p* = 0.002.

*Post hoc* tests with Bonferroni correction for ‘Flashed Schematic Eyes’ paradigm revealed that peak amplitudes were larger at parietal sites (7.09 ± 0.82 μV) in comparison to the central sites (5.26 ± 0.44 μV, p = 0.038). For ‘Flashed Face—Eye position change’ the amplitudes were higher at parietal sites (8.33 ± 0.93 μV) in comparison to the central sites (3.93 ± 0.40 μV, p < 0.0001) and frontal sites (3.99 ± 0.52μV, p = 0.004). For ‘Flashed Face—Eye and Head position change’ we observed greater P300 peak amplitudes at parietal sites (9.54 ± 1.22 μV) in comparison to the central sites (4.49 ± 0.62 μV, p < 0.0001) and frontal sites (3.78 ± 0.65 μV, p = 0.003). The analysis showed equivalent hemispheric responses, which are consistent with the expected symmetry from more conventional P300 paradigms.

For the animated paradigms (‘Animated 3D body—gaze change in 1 avatar’ and ‘Animated 4 avatar environment—gaze change in 4 avatars’) the *post hoc* tests confirmed an unexpected asymmetry in the amplitude distribution. For ‘Animated 3D body—gaze change in 1 avatar’ paradigm the P300 peaks amplitudes were higher at right (6.98 ± 0.65 μV) sites compared to left (5.01 ± 0.44 µV, *p* < 0.0001) sites. Also for ‘Animated 4 avatar environment—gaze change in 4 avatars’ the amplitudes were superior at right (4.54 ± 0.45 μV) electrode positions compared to Left (3.34 ± 0.36 μV, *p* = 0.002) sites. The amplitudes were superior at Parietal sites (5.41 ± 0.542 μV), comparing to Central (3.65 ± 0.40 μV, *p* = 0.001) and Frontal areas (3.77 ± 0.39 μV, *p* = 0.001). In sum, the P300 peak amplitudes of the animated paradigms (‘Animated 3D body—gaze change in 1 avatar’ and ‘Animated 4 avatar environment—gaze change in 4 avatars’) were significantly higher at right sites suggesting that a new component was superimposed to P300 signals in these social animated stimuli. Grand-averages concerning these paradigms are shown in Fig. 5 and Fig. 6, respectively.

Concerning latencies, Friedman tests of the mean latencies at the defined areas (frontal—F3, Fz, F4; central—C3, Cz, C4; parietal—P3, Pz, P4) and locations relative to midline (left—F3, C3, P3; right—F4, C4, P4) showed no effects of area and location for ‘Flashed Schematic Eyes’ paradigm (χ^2^(2) = 2.471, *p* = 0.291, χ^2^ (1) = 0.059, *p* = 0.808).

For ‘Flashed Face—Eye position change’ the area effects were significant (χ^2^(2) = 8.588, *p* = 0.014) but location effects were not observed (χ^2^ (1) = 2.882, *p* = 0.090).

For ‘Flashed Face—Eye and Head position change’ paradigm neither area effects (χ^2^(2) = 3.294, *p* = 0.193), nor location effects were significant (χ^2^ (1) = 0.250, *p* = 0.617).

The ‘Animated 3D body—gaze change in 1 avatar’ condition showed significant differences between areas (χ^2^ (2) = 9.882, *p* = 0.007). Location effects were not observed (χ^2^ (1) = 0.059, *p* = 0.808). We observed area effects in the ‘Animated 4 avatar environment—gaze change in 4 avatars’ paradigm (χ^2^(2) = 6.706, *p* = 0.035), but not location effects (χ^2^ (1) = 1.471, *p* = 0.225).

The *post hoc* analysis with Wilcoxon signed-rank tests with Bonferroni correction were applied, resulting in a significance level set at *p* < 0.017. For ‘Flashed Face—Eye position change’ the *post hoc* analysis revealed faster latencies at Parietal areas (374.78 ± 107.38 ms) comparatively to Central (505.65 ± 164.42 ms, Z = -2.675, *p* = 0.007) and Frontal (566.96 ± 201.55 ms, Z = - 2.438, *p* = 0.015) sites. For the ‘Animated 4 avatar environment—gaze change in 4 avatars’ and ‘Animated 3D body—gaze change in 1 avatar’ there were no significant differences in any of the mean latencies of the defined Areas.

**Fig 7:**
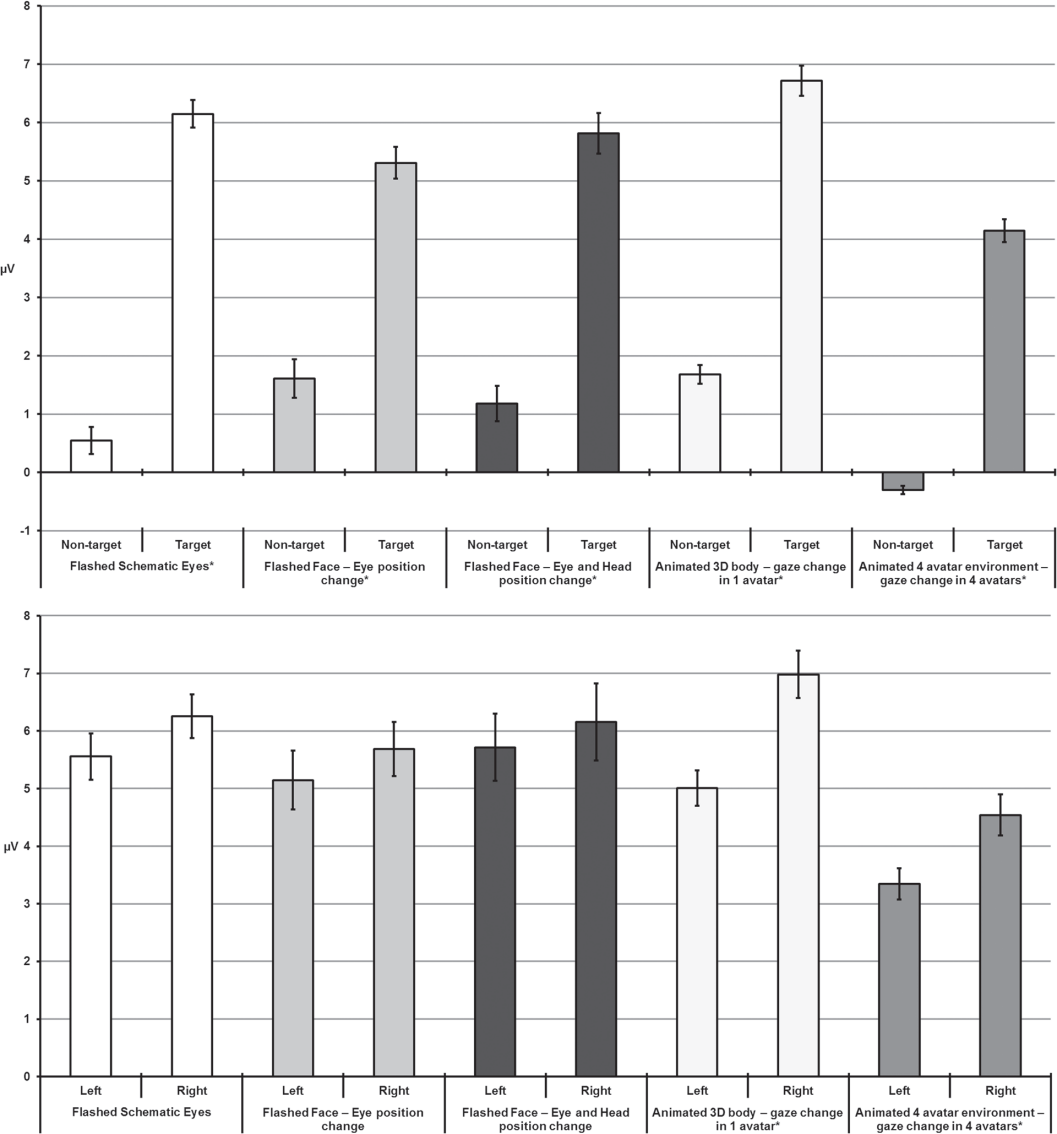
Summary of results. Top: target and non-target waveform amplitudes comparison. Bottom: P300 amplitudes were significantly higher in the right hemisphere for realistic animations (‘Animated 3D body—gaze change in 1 avatar’ and ‘Animated 4 avatar environment—gaze change in 4 avatars’). Error bars are the standard error of the mean.

Fig. 7 provides an overall summary of the main results reported in this study.

All participants were able to detect the 10 target events of each block in more than 99% of the cases. We had already shown that no detections imply the absence of a P300 matching the absence of behavioural report [60]. Therefore, the potential confound of no detection of target events is not present in our experiment.

We also performed control analyses comparing conditions whereby avatars gaze either to the left or to the right. Amplitude values for both types of gaze were virtually identical and were therefore not significantly different.

### Waveform classification

**Fig 8:**
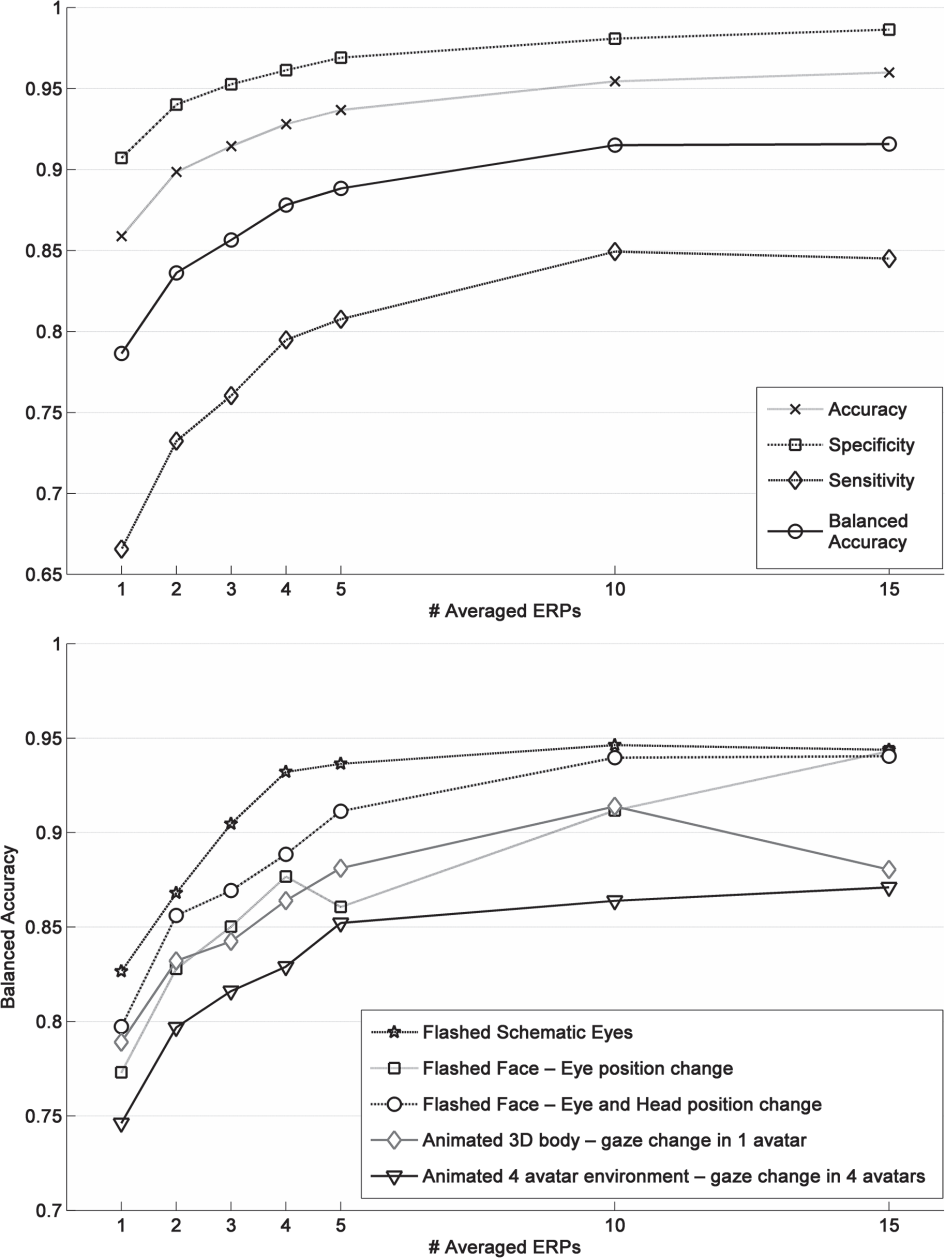
Classification results. Top: Results of several metrics with all paradigms averaged, for the different number of trials used. Bottom: Comparison of balanced accuracy between paradigms for the different number of events average.

Significant classification performance was found already at the signal trial level (see Fig. 8). The metrics used were accuracy (TP + TN) / N, specificity TN / (TN + FP), sensitivity TP / (TP + FN) and balanced accuracy (SP + SS) / 2 (TP, TN, FP, FN, SP, SS and N as True Positives, True Negatives, False Positives, False Negatives, Specificity, Sensitivity and total number of events, respectively). Permutations tests for each subject and paradigm, yielded p-values bellow 0.05 in 99,9% of the cases which means that the classifier performs well and is reliable. An improvement in classification performance was observed, as expected, when increasing the number of averaged EEG single-trial epochs due to the noise reduction effect of averaging. Yet, this increase is no longer relevant after 5 averaged epochs because of the probable loss of relevant information in averaging and the decreasing of the dataset size. As expected, classification results are worse but still significant for animated paradigms due to the complexity of the scene. The results are presented in Fig. 8.

Classification results comparison between stimuli conditions revealed a significant main effect (F(4, 80) = 2.483, *p* = 0.050). *Post hoc* analysis with Bonferroni correction showed that classification results were significantly better for ‘Flashed Schematic Eyes’ (0.91 ± 0.02) comparing to the more complex ‘Animated 4 avatar environment—gaze change in 4 avatars’ conditions (0.82 ± 0.19, *p* = 0.039).

## DISCUSSION

The goal of the present study was to study and identify the neurophysiological correlates of attention to realistic social scenes which degree of complexity was defined in a defined ordinal manner (Table 1), taking into account the number of items in the scene (including multipart social objects), elements that define the trajectory of social object and presence of movement. This was attempted at a single or near single-trial level, which would potentially allow pinpointing complex directed attention responses at the single event level. We found an oddball response for all the conditions and, importantly, we proved that the processing of realistic multi-agent actions (that can or not be interpreted as social) can be detected in human oddball responses both in average responses and at the single-trial level.

We found an oddball response for all the tested paradigms, irrespective of their complexity. Our waveforms analysis revealed a distinct P300-like waveform when it was elicited by animated stimuli representing realistic gestures. This signal does differ from the classic P300 by being right lateralized, which is not explained by low level features of the stimuli, given that it was neither found in our study, using the simpler paradigms, nor reported in the literature (see below discussion of potential confounds).

We hypothesize that the right lateralization is due to high level characteristics introduced by the realism of the animated paradigms, such as the reflexive attention generated by social gaze orientation. This hypothesis is supported by recent findings in fMRI studies [61–63] about the regions involved in gaze orienting. These studies described the influence of the right STS and other regions in processing of dynamic social attention cues (for a review see [48,64].

There is solid evidence for hemispheric asymmetries underlying the domain of social perception(for a review see [65]. Our results are consistent with the idea that the neural substrates of the perception of gaze, faces and related gestures are characterized by a general pattern of right-hemispheric functional asymmetry. It has been postulated that such substrates might benefit from other aspects of hemispheric lateralization in affective processing, instead of constituting an *isolated specialization* for social information. Individual recognition or social judgment is prioritized by the human brain and may benefit if it were localized only in one hemisphere [65]. Even simple face detection is already lateralized to the right hemisphere [66,67]. Processing of facial expressions of emotion, which is also relevant for social cognition, is known to be lateralized [68,69].

Concerning social cognition, gaze plays a central role in decoding others’ attention, goals and intentions, supporting the idea of a right-hemispheric bias for eye gaze perception. Hemispheric asymmetries in the neural substrates of gaze perception are present even in domestic chicks [70] and confirmed by human data [71]. In the latter work it was shown that eye gaze is processed better when presented in the left visual field. Following this idea, a right hemisphere involvement in social responses seems to be well-established among all vertebrates studied so far [72,73].

Biological motion was also a feature in our displays, in particular in the more complex animated versions. These stimuli are of evolutionary importance since social animals, such as humans, take decisions based on the interpretation of the actions of others. Saygin in [74] examined 60 unilateral stroke patients, and found no evidence suggesting lateralization for basic biological motion perception. Nevertheless, Pelphrey and colleagues in [75] found such a lateralization when studying perception of naturalistic social movements in complex context, which led Saygin et al. to infer that the right lateralization of biological motion perception may be explained by the ‘social’ aspects elicited by human motion rather than by body movement *per se*. It is demonstrated that BOLD response to dynamic faces is higher than to static faces in right STS [38]. Puce et al. in [44], described higher right STS activation during perception of moving eyes and mouth within a face. This is consistent with our results, since lateralization emerges for the more complex displays. Our data support the idea that social gaze orientation characteristics coupled to the ecological nature of our conditions elicited an increased asymmetric temporo-parietal response that adds to the reflexive attentional processes inherent to the ‘oddball’ structure of the paradigm.

Altogether the available evidence indicates that social orienting relies on asymmetric cortical mechanisms. Future studies should elucidate the nature of the realistic social processing network model underlying the identified neurophysiological component. Anyway, our results suggest that these signals are highly generalizable to realistic oddball contexts.

One could argue that the P300-like ERP lateralization is not related to the complexity of the stimulus presented, but to the direction where in the target stimuli the gaze is directed. It should be noted that both viewer and avatar frames of reference (left-right reversed) are distinct and simultaneously present, which makes predictions based on gaze direction not obvious (and if present would mask rather than emphasize our results). In any case, our new data and analyses comparing conditions where avatars gaze either to the left or to the right revealed that the amplitude values for both types of gaze were virtually identical. This potential confound is therefore ruled out under the conditions of the experiment (where both viewer and avatar frames of reference are distinct and simultaneously present).

An important validation of the relevance of these oddball signals was their data driven identification with high fidelity with few or even single-trials. The success of single-trial classification of P300 signals evoked by realistic gestures is important not only in cognitive neuroscience but also for the potential development of clinical applications including BCIs. Since cognitive processes depend critically on the specific situational context in which a subject is embedded [50], we can use this kind of stimuli to create realistic, structured and efficient models of social interactions that can be detectable with excellent temporal resolution. It potentiates the use of BCIs in clinical applications of adaptive social behaviour in normal subjects and disorders of social cognition such as in autism [11,51].

The usage of 16 electrodes can be viewed as a potential limitation of this study, but on the other hand the identification of these neural signals with very few electrodes at the single-trial level reinforces the idea that it is possible to use these signals as markers of attention within complex social events/scenes in BCI applications, with as few electrodes as possible. Further work should be made to find the best features that define this signal and the electrode positions that are best suited to provide such features. With this, one might drastically reduce the number of electrodes to use in clinical applications such as in autism, which will reduce the preparation time of the sessions, usually one of the major drawbacks of EEG applications in clinical BCI.

To conclude, we have shown that realistic animated oddballs with social content generate a specific response that can be successfully classified even at the single-trial level. We verified that this specific response is right lateralized for more complex scenes. We do believe that this work paves the way to study social cognition at the single or near single-trial and opens the door for future forms of cognitive training in diseases of social cognition.
